# FHF-independent conduction of action potentials along the leak-resistant cerebellar granule cell axon

**DOI:** 10.1038/ncomms12895

**Published:** 2016-09-26

**Authors:** Katarzyna Dover, Christopher Marra, Sergio Solinas, Marko Popovic, Sathyaa Subramaniyam, Dejan Zecevic, Egidio D'Angelo, Mitchell Goldfarb

**Affiliations:** 1 Department of Biological Sciences, Hunter College of City University, 695 Park Avenue, New York, New York 10065, USA; 2 Graduate Center of City University, Molecular, Cellular and Developmental Biology Subprogram, 365 Fifth Avenue, New York, New York 10016, USA; 3 Graduate Center of City University, Neuroscience Subprogram, 365 Fifth Avenue, New York, New York 10016, USA; 4 Brain Connectivity Center, C. Mondino National Neurological Institute, Via Forlanini 6, Pavia 27100, Italy; 5 Department of Cellular and Molecular Physiology, Yale University School of Medicine, 333 Cedar Street, New Haven, Connecticut 06510, USA; 6 Department of Brain and Behavioral Science, University of Pavia, Via Forlanini 6, Pavia 27100, Italy

## Abstract

Neurons in vertebrate central nervous systems initiate and conduct sodium action potentials in distinct subcellular compartments that differ architecturally and electrically. Here, we report several unanticipated passive and active properties of the cerebellar granule cell's unmyelinated axon. Whereas spike initiation at the axon initial segment relies on sodium channel (Na_v_)-associated fibroblast growth factor homologous factor (FHF) proteins to delay Na_v_ inactivation, distal axonal Na_v_s show little FHF association or FHF requirement for high-frequency transmission, velocity and waveforms of conducting action potentials. In addition, leak conductance density along the distal axon is estimated as <1% that of somatodendritic membrane. The faster inactivation rate of FHF-free Na_v_s together with very low axonal leak conductance serves to minimize ionic fluxes and energetic demand during repetitive spike conduction and at rest. The absence of FHFs from Na_v_s at nodes of Ranvier in the central nervous system suggests a similar mechanism of current flux minimization along myelinated axons.

## Abstract (web summary)

FHFs are known to regulate voltage-gated sodium channels (NaVs). Here, the authors compare the role of FHFs in cerebellar granule cell propagation, and find NaVs in the distal axon function independently of FHFs, allowing for faster inactivation rates and reducing energy demands during repetitive spiking.

## Graphical abstract

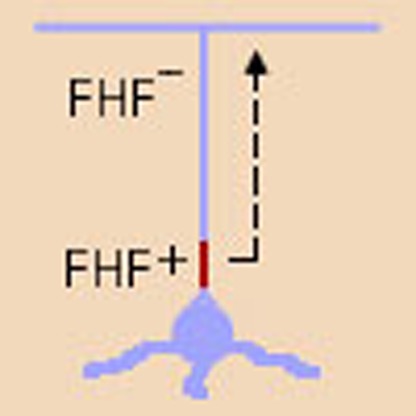

Neurons in the central nervous system (CNS) generate action potentials at the axon initial segment (AIS) in response to polysynaptic and intrinsic somatodendritic currents that traverse the soma and hillock and depolarize the AIS membrane to spike threshold^1^,^2^,^3^. Somatic capacitance extends the depolarization phase of excitatory postsynaptic potentials (EPSPs) to several milliseconds, and the slower EPSP decline phase allows for summation of successive excitatory inputs over longer timeframes. These temporal features necessitate the protection of voltage-gated sodium channels clustered in the AIS from inactivation during depolarization phases preceding spike threshold. In cerebellar granule cells (GrCs), cytoplasmic fibroblast growth factor homologous factors (FHFs) bound to sodium channels play an essential role in intrinsic excitability by raising the voltage dependence and slowing the rate of sodium channel inactivation as well as accelerating channel recovery on repolarization^4^,^5^. FHF modulation of fast inactivation has been shown to apply to several neuronally expressed sodium channel isoforms, including Na_v_1.1, Na_v_1.2 and Na_v_1.6 (refs 6, 7, 8, 9).

In contrast, regenerative axonal spike conduction may not benefit from a comparable tuning of sodium channels distributed more distally along the axon and parallel fibres. The 25–30-fold greater surface-to-volume ratio of a GrC's 150 nm diameter parallel fibres compared with its 5 μm diameter soma demands that a single action potential peaking at 60 mV generate a minimum 0.35 mM increase in intracellular sodium ion concentration along the axon and parallel fibres, assuming that all sodium influx were to be matched by outward capacitive current. If distally situated sodium channels were slow to inactivate, allowing for significant temporal overlap between the open states of voltage-gated sodium and potassium channels, sodium influx per spike could be far greater, and conduction of high-frequency spike trains would create a greater energy burden for rapid Na^+^/K^+^ pumping and ATP synthesis.

These theoretical considerations motivated our investigation of axonal conductance properties using biochemical, genetic, optical and computational tools. We show here that spike conduction along the GrC axon occurs in an FHF-independent manner predicted to minimize current fluxes. These active properties combined with an unexpectedly low-leak conductance serve to minimize energy expenditure within the ultra-thin axon.

## Results

### Most sodium channels on GrC distal axon lack FHF

**Figure 1:**
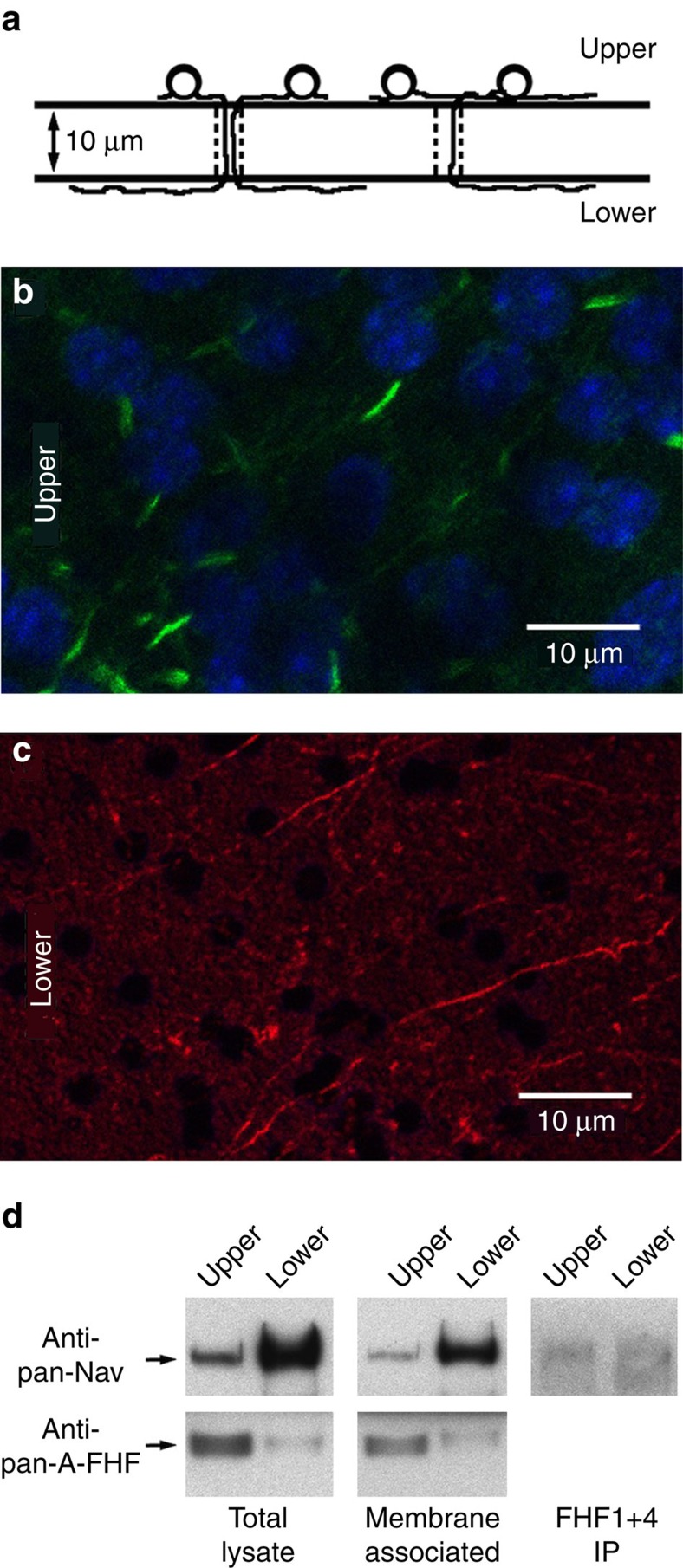
Limited association of FHFs with sodium channels on distal axons of cultured GrCs. (**a**) Schematic depiction of culture system. Granule cells dissociated from juvenile mice (P8) are seeded onto the upper surface of hanging culture filter membranes (10 μm thick, 3 μm diameter pores) and cultured for 25 days. Growing axons can traverse pores and continue extension along lower surface. (**b**) Confocal fluorescence imaging of an upper surface showing TOPRO iodide-stained nuclei (pseudocoloured blue) and short ∼5 μm AISs visualized with anti-AnkyrinG monoclonal antibody (green). (**c**) Fluorescence imaging of a lower surface showing distal axons detected with neurofilament-A antibody (red) and lacking cell soma (blue). (**d**) FHF, Nav and FHF/Nav complexes in lysates of total cells and distal axons. Left; 20 μg protein extracted from upper surface (whole cell) and 10 μg protein extracted from lower surface (distal axons) were directly electrophoresed and immunoblotted for detection of sodium channels with pan-Na_v_ antibody and detection of all A-type FHFs with pan-A-FHF antibody. Center; live cultures were surface-biotinylated before extraction and streptavidin-agarose pull-down of labelled proteins, which were then immunoblotted for sodium channels and A-type FHFs. Right; equal amounts of whole cell (upper surface) and distal axon (lower surface) lysates were immunoprecipitated with a combination of antibodies specific for all isoforms of FHF1 and FHF4 followed by immunoblot detection of sodium channels. A much smaller fraction of sodium channels in distal axons show association with any of the FHFs tested. The corresponding uncropped immunoblots are shown in Supplementary Fig. 1.

A potential mechanism for accelerating inactivation of sodium channels along the axon is for channels to have limited association with FHF proteins. To test this hypothesis, we performed immunoblot analysis on isolated distal axons of GrCs obtained using a hanging filter culture system (Fig. 1a). Neurons plated on top of the filter project axons that can pass through pores (10 μm length, 3 μm diameter) and further extend on the lower surface. Immunofluorescence can detect the AIS and soma of neurons on the upper surface of the filter (Fig. 1b), but owing to the 5 μm length of the granule cell AIS^10^, axon processes on the lower surface are all distal in nature (Fig. 1c). Lysates prepared from scrapes of upper and lower filter surfaces were directly analyzed by immunoblotting using an FHF monoclonal antibody that recognizes an epitope common to the A-type isoforms encoded by all four *Fhf* genes^11^ and another monoclonal that detects all voltage-gated sodium channels^12^. Compared with upper-surface whole-cell lysates, the ratio of A-type FHF to sodium channels in lower-surface distal axon lysates is reduced 5–10-fold (Fig. 1d, left). This finding was reproducible in five independent preparations of lysates from hanging cultures. A similar result was obtained when analysis was restricted to surface membrane-associated proteins prepared by streptavidin-agarose capture of surface-biotinylated proteins (including sodium channels) (Fig. 1d, center). Furthermore, when lysates were immunoprecipitated with a mixture of antibodies that recognize all protein isoforms encoded by the *Fhf1* and *Fhf4* genes, a much smaller fraction of sodium channels was detected in the distal axon preparation compared with whole cells (Fig. 1d, right). Since the mouse *Fhf3* gene is only known to generate an A-type transcript (Mouse Genome Informatics) and the *Fhf2* gene is not expressed in the GrCs (Allen Brain Atlas), our analysis provides complete coverage of all expressed FHF isoforms and demonstrates that most sodium channels on granule cell distal axons are not associated with FHFs.

### FHFs are absent from nodes of Ranvier in the CNS

**Figure 2:**
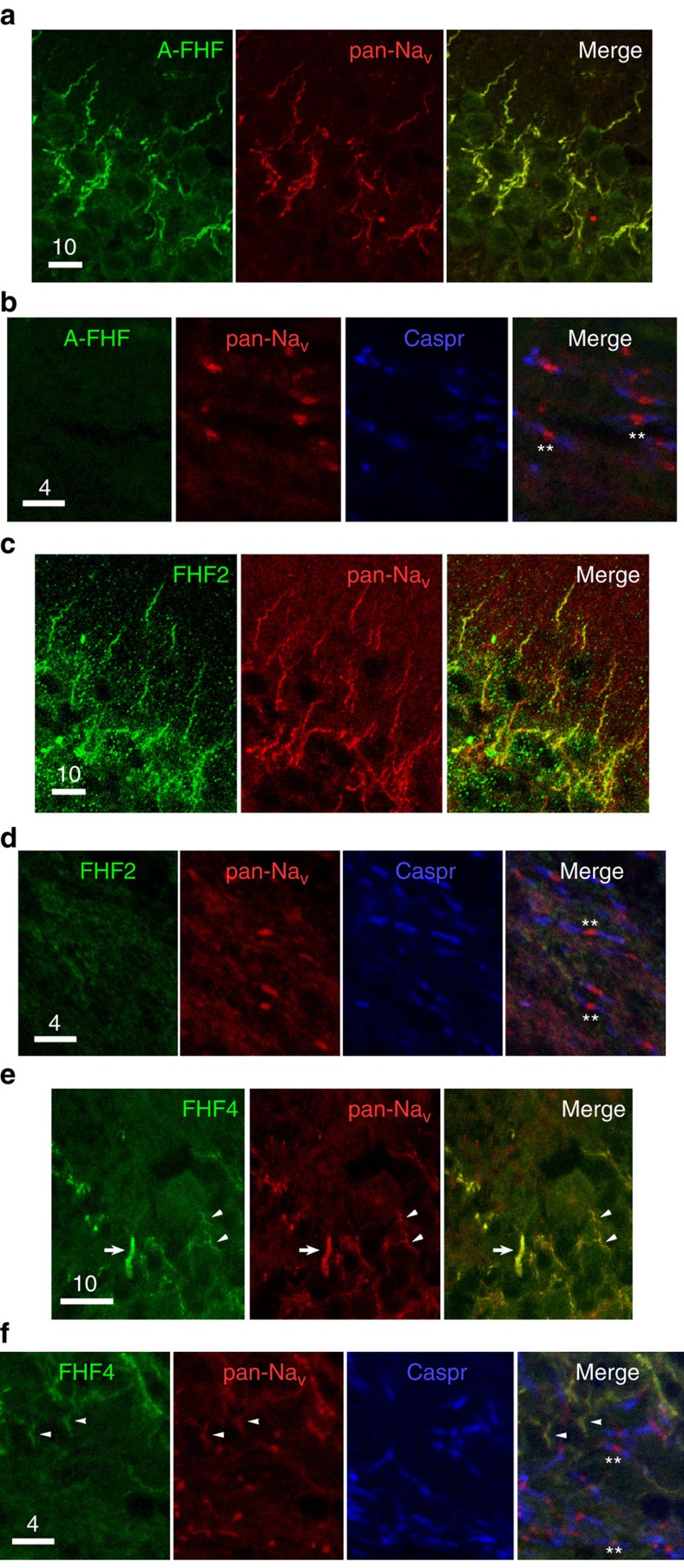
Preferential absence of FHFs from nodes of Ranvier along CNS-myelinated axons. (**a**) Immunofluorescence colocalization of A-type FHFs (green) and sodium channels (red) at hippocampal CA1 pyramidal neuron AISs. (**b**) Absence of A-type FHFs (green) from corpus callosal nodes of Ranvier visualized by sodium channels (red) flanked by paranodal Caspr (blue) (**). Further A-type FHF immunofluorescence is provided in Supplementary Fig. 2. (**c**) Colocalization of FHF2 (green) and sodium channels (red) at AIS of CA1 pyramidal neurons. (**d**) Absence of FHF2 (green) from corpus callosal nodes of Ranvier (red) flanked by paranodal Caspr (blue) (**). (**e**) Colocalization of FHF4 (green) and sodium channels (red) at a cerebellar Purkinje cell AIS (arrow) along with AIS of numerous granule cells (arrowheads). (**f**) FHF4 (green) is absent from nodes of Ranvier (red) in the cerebellar cortical white matter (**), while present in adjacent granule layer AISs (arrowheads). Scale bars, (**a**,**c**,**e**) 10 μm and (**b**,**d**,**f**) 4 μm.

In order to test whether FHFs are associated with sodium channels on distal axons in the CNS, we assayed for localization of FHFs to nodes of Ranvier along myelinated axons, as the low density and uniform distribution of sodium channels along unmyelinated axons precluded potential detection of associated FHFs on unmyelinated axons. A-type FHFs were readily detected at the AIS of virtually all large-diameter CNS neurons, including brain stem motoneurons and pyramidal neurons in cerebral cortex and hippocampus (Fig. 2a, and ref. 11). However, these FHFs were not seen on Na_v_-positive axonal nodes within the corpus callosum (representative nodes and Caspr-flanking paranodes in Fig. 2b) or cerebellar white matter (Supplementary Fig. 2). Similarly, antibodies to FHF2 proteins strongly stained the AIS of all hippocampal pyramidal neurons (Fig. 2c), but did not detect these proteins at nodes in the corpus callosum (representative nodes in Fig. 2d). Furthermore, FHF4 proteins were detected at the AIS of cerebellar Purkinje cells (Fig. 2e), as previously reported^13^, but were not detected at any nodes of Ranvier within the cerebellar cortical white matter, which bears the descending Purkinje cell axons along with ascending axonal projections (representative nodes in Fig. 2f). Hence, restriction of FHFs from distal axonal sodium channels is a feature common to both unmyelinated and myelinated CNS axons.

### FHFs are not required for GrC axonal spike conduction

The above biochemical analysis suggested that action potential conduction along granule cell axons does not require sodium channel modulation by FHFs. To test this prediction, we prepared GrC cultures from wild-type (WT) and *Fhf1*^−/−^*Fhf4*^−/−^ mice^4^. Cultured neurons bear between one and four short thick dendrites and a thin axon extending over 1 mm, often with one or more major collaterals and varicosities spaced an average of 5 μm apart (Supplementary Fig. 3A). Whereas cultured WT neurons fired repetitively when injected with continuous positive current, cultured mutant neurons could only fire once or twice in response large current injection (Supplementary Fig. 3B,C), recapitulating the behaviour of these cells in acute brain slice preparations^4^. To study the properties of conducting action potentials, individual cells were each patched with a pipette containing the voltage-sensitive ANEPPS fluorophore JPW3028 (ref. 14). Following 15 min of filling by passive diffusion, the pipette was removed and the dye was allowed to permeate the axon for 60 min before re-patching and recording. Voltage clamping of the electrically compact somatodendritic compartment^10^ showed that dendritic fluorescence and membrane voltage display a linear relationship over the span from −100 to +60 mV (Supplementary Fig. 4).

**Figure 3:**
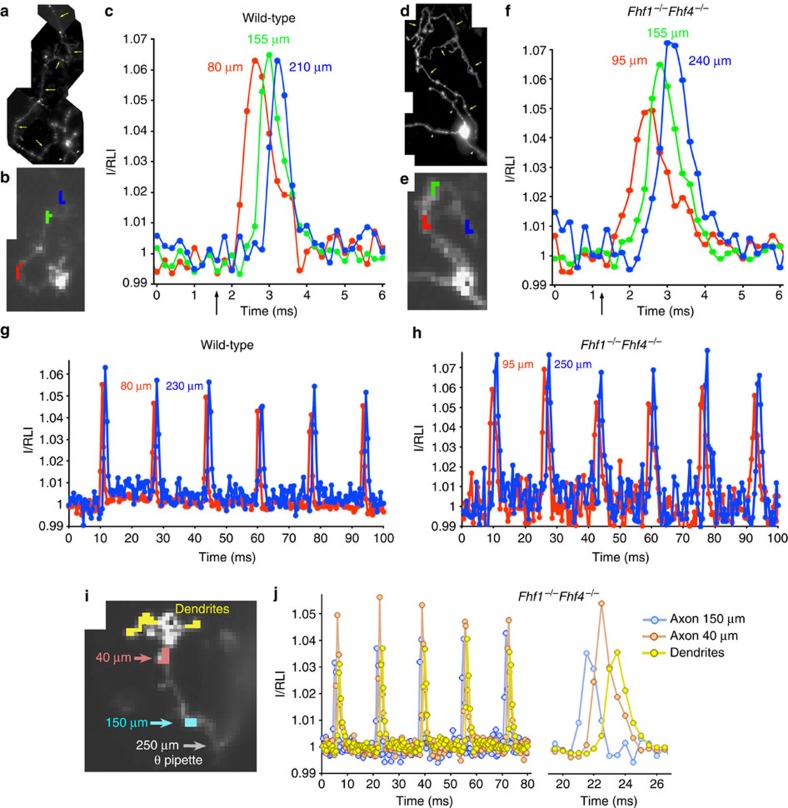
Fluorescent imaging of action potential conduction along axons of WT and *Fhf1*^−/−^*Fhf4*^−/−^ cultured GrCs. (**a**) High-resolution image composite for WT neuron filled with voltage-sensitive fluorophore JPW3028. Dendrites (arrowheads) and axon (yellow arrows) are indicated. (**b**) Same cell as in A photographed at 0.2 ms exposure with high-speed, low-resolution camera used for dynamic fluorescence imaging. Coloured pixels are regions along axon monitored during spike conduction. (**c**) Spike conduction along axon of cell in **a**,**b** in response to 0.2 ms 2 nA somatic current pulse (arrow). Fluorescence was sampled at 5 kHz in regions indicated by colours corresponding to **b**. Stimulation induced spike with Δ*F* of 6.5% that conducted down axon at ∼0.2 mm ms^−1^. Data shown are the average from 40 stimulus trials. I=fluorescence intensity, RLI=resting light intensity at the sampled region. (**d**) High-resolution image composite for *Fhf1*^−/−^*Fhf4*^−/−^ neuron, with dendrites (arrowheads) and axon (yellow arrows) indicated. (**e**) Same mutant cell imaged at high-speed and low-resolution. (**f**) Spike conduction along mutant cell axon (**d**,**e**) in response to somatic current pulse (arrow) was indistinguishable in amplitude (beyond 150 μm) and conduction velocity compared with spike from WT cell (**c**). (**g**) 60 Hz spike conduction along WT axon. The JPW3028-filled neuron was injected in the soma with biphasic current pulses (700 pA for 1 ms followed by −700 pA for 1 ms) at 60 Hz to induce spike train. Fluorescence was sampled at 2 kHz at different distances along the axon, and data averaged over 15 trials. The spike train faithfully conducted over 230 μm. (**h**) A total of 60 Hz spike conduction along *Fhf1*^−/−^*Fhf4*^−/−^ axon. The mutant cell was subjected to same analysis as in **g**, and showed faithful transmission of the spike train over 250 μm. (**i**,**j**) Antidromic spike conduction along axon of *Fhf1*^−/−^*Fhf4*^−/−^ cell. A mutant granule cell axon was stimulated at 60 Hz at a point 250 μm from the soma, and fluorescence was analyzed at proximal regions, as indicated in high-speed still image (**i**). All spikes back-propagated towards soma and were detectable in dendrites (**j**).

Individual action potentials were triggered by a 0.2 ms 2 nA current pulse delivered through the patch pipette, and change in fluorescence intensity was monitored at a frame rate of 5 kHz. Dye permeation down the axon was sufficient to allow fluorescence measurements at up to 250 μm from the soma. Figure 3a shows a composite of high-resolution images from a WT cell, while Fig. 3b is a high-speed, low-resolution image of the same cell taken at lower magnification during voltage-sensitive dye recording. Temporal fluorescence data from spatially-averaged axon pixels highlighted in Fig. 3b are shown in the graph in Fig. 3c. The current pulse triggered an action potential-related optical signal with peak height of 6.5% resting light intensity, full width at half height of ∼0.7 ms at a point 155 μm along the axon, and conduction velocity of ∼0.2 mm ms^−1^ (Fig. 3c), consistent with spike conduction velocities along ascending axon^15^,^16^ and parallel fibres^17^,^18^ of these neurons *in situ*. A movie of spike generation and conduction visualized with voltage-sensitive fluorescent dye (Supplementary Movie 1) illustrates spike initiation in a proximal axonal region within 10 μm from the soma followed by both backpropagation to the soma and forward propagation along the axon and spike bifurcation at axonal branchpoints. An *Fhf1*^−/−^*Fhf4*^−/−^ (KO) granule cell conducted an axonal spike with similar velocity, amplitude, and width (Fig. 3d–f). A cohort of WT (*n*=9) and KO (*n*=10) granule cells showed small, insignificant differences in axonal spike peak amplitude (WT 7.1±0.3% Δ*F* versus KO 6.5±0.2% Δ*F*; *P*>0.1) and conduction velocity (WT 0.20±0.02 mm ms^−1^ versus KO 0.17±0.01 mm ms^−1^; *P>*0.2).

In order to test for faithful conduction of action potentials generated at high frequency, dye-filled neurons were stimulated in current clamp with biphasic current pulses (positive current 700 pA for 1 ms followed by equal negative current for 1 ms) at 60 Hz. The negative phase of each current pulse helped drive the somatic and proximal axonal membrane voltage quickly back near resting potential, facilitating the recovery from inactivation of proximal sodium channels even in mutant neurons lacking FHFs. Using this stimulation protocol, all tested WT (*n*=6) and KO (*n*=9) cells were capable of conducting a 60 Hz train of action potentials down their axons (examples in Fig. 3g,h).

We further tested whether a KO granule cell can conduct a train of antidromic spikes in response to pulsed stimulation of the distal axon. As shown in Fig. 3i,j, a 60 Hz train of spikes induced in the axon at a distance of 250 μm from the soma can be conducted back to the neuron's soma and dendrites. These data confirm that FHFs reside on sodium channels in the proximal axon where they promote spike initiation while being largely excluded from more distal channels that mediate spike conduction in an FHF-independent manner.

### Passive properties of the GrC distal axon

**Figure 4:**
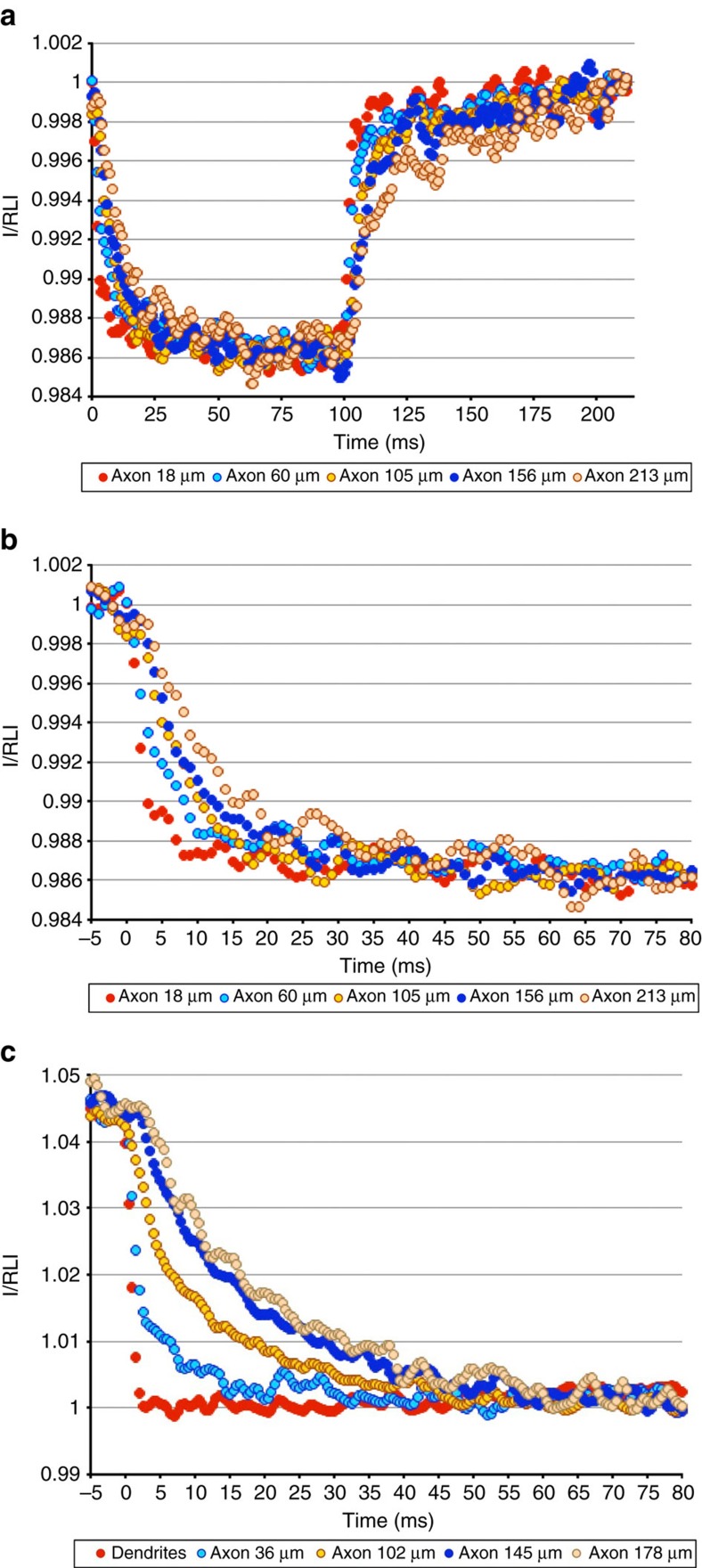
Passive charging of cultured GrC axons in response to somatic voltage clamp. (**a**) Axonal response to somatic hyperpolarization. A WT neuron filled with JPW3028 was patched on the soma, held at −60 mV and subjected to a 100 ms −100 mV step followed by step return to −60 mV. Fluorescence was sampled at 2 kHz, and data were averaged over 50 trials. At each axonal position, fluorescence intensity (I) was normalized to that position's resting light intensity (RLI). The voltage step induced very similar fluorescence changes at points ranging from 18 to 213 μm, suggesting comparable charging and limited leak conductance. (**b**) Expanded time scale of data in **a**, showing slower charging of axon as function of distance from soma. (**c**) Passive axonal response to large somatic voltage change. A WT neuron filled with JPW3028 was patched on the soma in the presence of tetrodotoxin, tetraethylamine, 4-amino purine and cadmium to inhibit voltage-gated sodium, potassium and calcium channels. The soma was stepped from −80 to +80 mV for 100 ms followed by return to −80 mV and images collected at 2 kHz intervals. Fluorescence at different points along axon are shown for the repolarization step initiating at *t*=0 ms. All points along axon ranging from 36 to 178 μm undergo very similar change in fluorescence, with longer charging times as function of distance from soma. Data shown are the average of 50 trials.

An understanding of the passive cable properties of the granule cell axon could help answer two questions related to spike conduction. First, what is the leak conductance along the axon that will influence both the sodium influx needed to generate a conducting spike and the velocity of spike conduction? Second, is passive charging consistent with an axonal diameter similar to the 150 nm diameter of parallel fibres *in vivo*^19^? To address these questions, fluorescence changes in JPW3028-filled axons were monitored in response to voltage-clamped step commands elicited at the soma. Figure 4a shows axonal fluorescence measurements during a 100-ms step from −60 to −100 mV and a return to −60 mV thereafter. All measured points along the axon, ranging from 18 to 213 μm from the soma, experienced very similar fluorescence changes, reflecting similar voltage changes and implying a very low axonal leak conductance. The same data presented on an expanded time axis (Fig. 4b) shows that axonal charging time is slower as a function of distance from soma, as expected. To better visualize charging times along the axon in voltage clamp, a step from +80 to −80 mV was employed in the presence of voltage-gated channel inhibitors. As shown in Fig. 4c, fluorescence changes in response to the large voltage step were very similar at all measured points along the axon, with charging times increasing as a function of distance. These fluorescence data can be used in conjunction with computation modelling (see below) to achieve better quantitative understanding of the granule cell axon's passive properties.

### Computational modelling of the GrC axon

**Figure 5:**
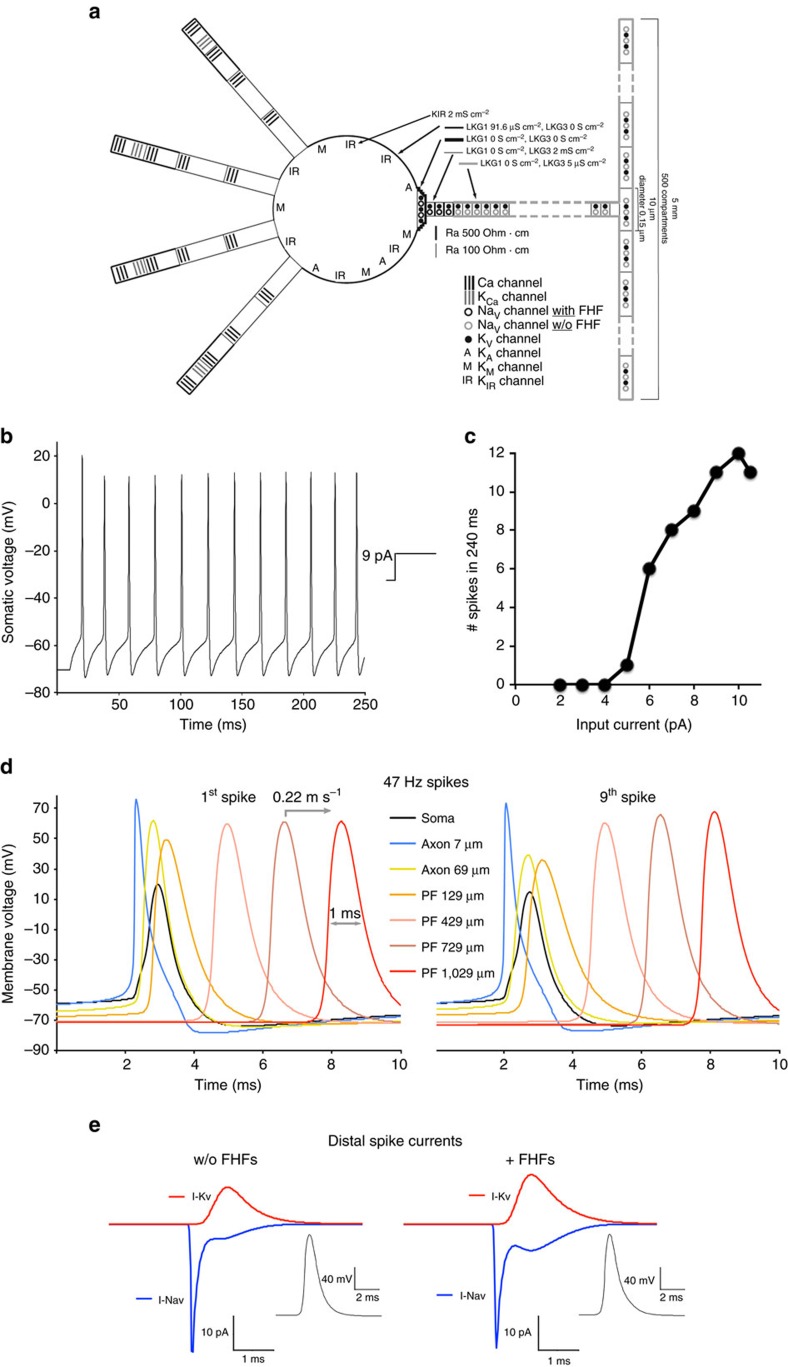
Revised GrC computational model predicts energy benefit of FHF-independent spike conduction. (**a**) Schematic of revised model. Clustered sodium channels at hillock and AIS are modelled with associated FHFs (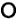), while low-density sodium channels on distal axon and parallel fibres are modelled without FHFs (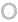) (see Supplementary Fig. 5 for Na_v_ simulations +/− FHF). Fibre leak conductance (LKG3) is set to 2 mS cm^−2^ at the AIS (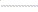) and to 5 μS cm^−2^ along distal axon and parallel fibres (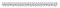). Somatic leak conductance (LKG1) combines with inward rectifying potassium conductance (KIR) to set somatic resting membrane potential to −70 mV. The AIS also has elevated axial resistance (500 Ohm*cm, 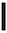) compared with all other cellular compartments (100 Ohm*cm, 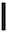). (**b**) Somatic spikes simulated in response to 10 pA somatic current injection. Spike frequency is similar to that described in the earlier version of the GrC model^10^. (**c**) Current-to-spike relationship. Plot of spike number over 240 ms during 2–10 pA simulated current injections. (**d**) High-frequency spike initiation and propagation. Voltage in soma and axonal and PF compartments (distance from soma indicated) are plotted in response to simulated 8 pA somatic injection generating a 47 Hz spike train. Spikes initiating at AIS conduct down axon and parallel fibres and back-propagate to soma. Parallel fibre spike conduction velocity (0.22 mm ms^−1^) and 50–50% width (1.0 ms) are within 10% of fluorescence measurements (Fig. 3c,e). (**e**) Parallel fibre spike currents modelled with or without associated FHFs. Left; sodium (blue) and potassium (red) currents in a parallel fibre compartment during a conducting spike (inset) as in **d**. On the basis of current fluxes, axoplasm volume, and equation 1 C=10 μmol cations, calculated Na^+^ influx and K^+^ efflux from parallel fibre are 0.59 mM per spike. Right; parallel fibres were remodelled with all sodium channels associated with FHF, and Na_v_ and K_v_ densities were adjusted to preserve shape and amplitude of the conducting spike (inset). Slower Na_v_ inactivation due to FHF increased temporal overlap between Na_v_ and K_v_ currents, thereby increasing calculated Na^+^ influx and K^+^ efflux (0.86 mM) per spike.

The empirical data described above has been used to update a prior multi-compartment model of the GrC^10^. The revised model scheme is shown in Fig. 5a.

The limited decay of fluorescence change over distance during passive charging implies a very low axonal leak conductance. To estimate the maximal possible axonal leak conductance density, voltage clamp simulations were conducted by applying a hyperpolarizing voltage step in the parallel fibre and measuring the voltage changes along the fibre after 100 ms. Leak conductance densities up to 5 μS cm^−2^ have small effects on fibre charging, while higher leak causes a greater fall-off of charging over a 200 μm distance (Supplementary Fig. 5A). As our empirical data shows that somatic voltage steps induce axonal fluorescence changes that diminish no more than 5% over a 200 μm distance (Fig. 4), axonal and parallel fibre leak was set at 5 μS cm^−2^ (Fig. 5a). This axonal leak density is less than 1% that in somatodendritic membrane, where inwardly rectifying potassium channels (K_IR_, 2 mS cm^−2^) are the major carrier of somatic leak current (Fig. 5a)^10^. Given the 26-fold greater surface-to-volume ratio in the axon and parallel fibres compared with the granule cell soma, low-distal leak affords substantial energy benefit and lessens energy consumption previously predicted for maintaining the granule cell resting potential^20^.

There is a disparity between the change in axonal fluorescence intensity during a conducting spike (Δ*F*=6.8±0.2% , *n*=19; examples in Fig. 3) compared with the change in fluorescence induced by passive somatic voltage commands (40 mV step gave Δ*F*=1.55±0.2%, *n*=6; examples in Fig. 4). Given the linear relationship between Δ*F* and Δ*V* (Supplementary Fig. 4), the fluorescence data would suggest a spike amplitude greater than 170 mV, which is untenable given a sodium ion reversible potential of ∼80 mV. This disparity was accommodated by assuming that a region of the axon proximal to distances of reliable measurement (<20 μm from soma) has higher leak conductance that acts as a partial current shunt. Accordingly, leak conductance in the AIS of the model (first three segments of axon spanning 7 μm, Fig. 5a) was set at 2 mS cm^−2^, comparable to somatic leak density.

Voltage clamp simulations run on cell models with axon diameter ranging from 100 to 300 nm all failed to produce passive axonal charging rates along the axon consistent with fluorescence measurements on several recorded cells (Supplementary Fig. 5B). By incorporating an elevated axial resistance into the AIS of a 150-nm diameter axon model^19^, a more accurate simulation of the passive charging was obtained (Supplementary Fig. 5C). Elevated resistance along the AIS may result from the documented dense packing of cytoskeletal fibrils in this specialized structure^21^,^22^.

The high-density voltage-gated sodium channels in the AIS were modelled as being associated with FHF proteins that favour excitability, while less dense channels on more distal axon and parallel fibres were modelled as lacking FHFs (Fig. 5a), consistent the data in Figs 1 and 3. For channels with or without FHFs, 12-state Markov models were employed with much slower activation and deactivation kinetics than described in several previous Na_v_ Markov models^10^,^23^ (see ‘Methods' section for details and necessary rationale). With these modifications, transient sodium current activation and inactivation kinetics (Supplementary Fig. 5D,G), voltage dependence of activation and inactivation (Supplementary Fig. 5E), and recovery rates from inactivation (Supplementary Fig. 5F) are good approximations to empirical data reported for granule cell sodium channels with or without FHFs in our earlier studies^4^ (Supplementary Table 1).

When positive current is continuously injected into the soma of the granule cell model, repetitive firing initiates in the AIS and each spike back-propagates to the soma and conducts down the axon and parallel fibres (Fig. 5b–d). Spike peaks at the soma are between 0 and +20 mV, consistent with granule cell recordings in cerebellar slices and primary culture^9^,^15^, while conducting spikes peak at +60 mV in the parallel fibres, consistent with spikes that have been recorded at presynaptic axonal termini^24^. As shown in Fig. 5d, conducting spikes display velocity (0.22 mm ms^−1^), width (1 ms 50%-to-50%), and absence of after-hyperpolarization consistent with spikes imaged using voltage-sensitive dye (Fig. 3). Due to the accelerated inactivation of FHF-free sodium channels along the axon and parallel fibres, the voltage-gated sodium and potassium currents for each conducting spike have little temporal overlap (Fig. 5e, left graph). In conjunction with negligible outward leak current, virtually all ionic currents can drive capacitive currents underlying rise and decline of the spike. For a 150-nm axon cable, the theoretical minimum ionic currents for driving an action potential peaking at +60 mV results in 0.35 mM sodium influx and equal potassium efflux. The model predicts sodium and potassium fluxes less than double the theoretical minimum (0.59 mM), which is comparable to prior model predictions of energy efficiency for granule cell conducting spikes^25^.

To assess the importance of accelerated distal sodium channel inactivation, we remodelled the distal axon and parallel fibres with sodium channels that bear FHFs, comparable to channels at the AIS^4^. Sodium and potassium channel densities were rebalanced to achieve a conducting spike waveform with shape and amplitude matching that simulated in the absence of FHF (Fig. 5e) (all parameters in ‘Methods' section and Supplementary Table 2). With FHFs present on sodium channels, there is substantially greater temporal overlap between voltage-gated sodium and potassium currents (Fig. 5e, right graph), resulting in ∼50% greater (0.86 mM) sodium influx and potassium efflux per spike than in the simulation where FHFs are excluded from distal sodium channels. These findings underscore the importance of spatially restricted FHF modulation of sodium channels along the axon, particularly during conduction of high-frequency spike trains.

## Discussion

Data reported here in conjunction with prior findings identify three zones along a GrC's unmyelinated axon having distinct reliance on FHF protein functionality. At the AIS, FHFs associate with sodium channels to delay onset of fast inactivation and accelerate recovery from inactivation, thereby promoting neuronal excitability^4^. As shown here, sodium channels beyond the AIS function in an essentially FHF-independent manner, thereby shortening the duration of the open-state phase during spike conduction and lessening energy burden. Recent data from other investigators describe a distinct role for FHFs at presynaptic terminals along axonal parallel fibres, promoting neurotransmitter release by enhancing glutamate vesicle loading via VGluT1 transport^26^. What mechanisms may enable the preferential delivery of FHFs to proximal sodium channels and still allow FHFs to act at distal presynaptic terminals? All FHF isoforms bear a common β-trefoil core domain that can interact with a cytoplasmic tail domain that is also highly conserved among all voltage-gated sodium channels^27^,^28^. The preferential association of FHFs with proximal sodium channels may result from other molecular constituents that stabilize FHF/Na_v_ interactions within the AIS. More simply, the presence of dense FHF affinity sites in the form of sodium channels at the AIS together with the AIS's diffusion barrier property^21^ may serve to limit FHF access to more distal axonal channels. FHFs are also known to associate with the kinesin-binding protein IB2 (ref. 29), providing a potential mechanism for microtubule-based transport of FHFs to presynaptic terminals for their contribution to neurotransmission.

The low axonal leak conductance in the GrC may serve several distinct functions. First, the total surface area of axonal and parallel fibre membranes is over 4,000 μm^2^, over 10-fold greater than the area of somatodendritic membrane. Without reduced fibre leak, maintenance of sodium and potassium concentration gradients across the plasma membrane at rest would require far greater energy consumption, as had been predicted previously^20^. Second, low axonal leak helps minimize sodium current density needed to conduct action potentials. Third, low-leak conductance accounts for documented long-range passive electrical transmission along this axon. Pugh and Jahr^30^,^31^ have demonstrated that molecular layer GABA transmission onto parallel fibre GABA_A_ receptors induces axonal depolarization that undergoes passive antidromic spread towards the soma resulting in enhanced somatic excitability of granule cells. In the absence of low axonal leak, this retrograde effect would be more limited or absent.

The physiological benefit afforded to the unmyelinated GrC axon may also apply to many CNS-myelinated axons, where FHFs are not detected on nodal sodium channels (Fig. 2; Supplementary Fig. 2). Shorter spike current duration in the absence of FHFs may reduce the needed leak conductance that serves as the primary carrier of outward current driving the nodal spike repolarization phase^32^. In contrast, myelinated sensory axons in the peripheral nervous system display prominent clustering of FHF2 at nodes of Ranvier^33^, indicating a fundamental difference in sodium channel modulation along these peripheral axonal fibres. Indeed, for larger diameter axons where energetics is a somewhat less serious imperative, FHF association with distal axonal sodium channels may be better tolerated and serve to regulate sodium conductance, action potential waveform, conduction velocity and synaptic transmission.

While our reported findings provide new insight into the ionic mechanisms and energetic burden in an unmyelinated CNS axon, two limitations of these studies serve as impetus for further investigation. First, dynamic fluorescence imaging was conducted here only at 25 °C. As temperature elevation will impact kinetics of voltage-gated channel state transitions as well as the conductance of open channels, the granule cell conducting spike waveform and underlying ionic currents at 37 °C will differ in ways that are not fully predictable without further recordings. Nonetheless, the energetic benefit of FHF-independent spike conduction due to accelerated sodium channel inactivation will still apply. Second, as our dynamic fluorescence measurements employed somatic loading of dye into cultured neurons, we were not able to assess the impact of en passant parallel fibre-Purkinje cell synapses on the passive and active properties of the granule cell axon and its multi-millimetre parallel fibres. The emerging technology of genetically engineered voltage indicators^34^,^35^ and their targeted expression to subsets of GrCs may allow for similar analysis of axon electrical properties in situ.

## Methods

### GrC culture

The husbandry and use of all mice followed protocols approved by the Hunter College Institutional Animal Care and Use Committee. GrC cultures were prepared from P8-P9 WT and *Fhf1*^−/−^*Fhf4*^−/−^ mouse pups as previously described^4^,^9^. Dissociated neurons were seeded onto either 12 mm coverslips for later recordings or onto the upper surface of submerged hanging culture membranes (3 μm pore diameter, 10 μm thick) (Catalogue PISP30R48, EMD Millipore). Coverslip cultures were maintained for 20–40 days for recordings, while hanging cultures were maintained 25 days before harvesting for protein analyses.

### Protein analysis of granule cells

To prepare lysates for protein detection, hanging culture inserts were washed and each side was separately scraped in ice-cold lysis buffer (20 mM Tris–HCl pH 7.4, 137 mM NaCl, 2 mM EDTA, 25 mM β-glycerophosphate, 2 mM pyrophosphate, 50 mM NaF, 10% glycerol, 0.1 mM sodium orthovanadate, 1 mM PMSF, 10 mg ml^−1^ aprotinin, 10 mg ml^−1^ leupeptin). Upper-surface scraped material was directly solubilized with 1% Triton X100, while lower-surface material was concentrated by high-speed centrifugation and then solubilized. Triton X100-treated samples were vortexed, maintained on ice for 15 min, and cleared of insoluble material by high-speed centrifugation. For some cultures, surface proteins were covalently biotinylated using sulfo-NHS-biotin (Catalogue 21217, ThermoFisher Scientific) before scrape harvesting as above. Biotinylated protein complexes were captured with streptavidin-agarose (Catalogue 20347, ThermoFisher). FHF immunoprecipitations were performed using a combination of 1 μg ml^−1^ custom rabbit anti-FHF1 (ref. 29) and mouse anti-FHF4 (N56/21, Catalogue 75–096, NeuroMab), both of which have been prior validated for immunoprecipitation competence. Streptavidin-agarose-captured, immunoprecipitated/protein G Sepharose-captured (Catalogue 17-0618, GE Healthcare), and total protein samples were electrophoresed through precast 4–20% polyacrylamide SDS gels (Catalogue 25204, ThermoFisher), transferred to PVDF membrane, and probed with 0.5 μg ml^−1^ mouse monoclonal antibodies to A-type FHF (N235/22, Catalogue 75-246, NeuroMab) and pan-sodium channel (K58/35, Catalogue S8809, Sigma Aldrich) followed by secondary peroxidase-conjugated antibodies (1:10,000, Catalogue 115-035-166, Jackson Immunoresearch) and enhanced chemiluminescence detection. Images in Fig. 1d were cropped for presentation. Full size images are presented in Supplementary Fig. 1.

For immunofluorescence, hanging culture membranes were fixed in 4% paraformaldehyde, permeabilized with 0.5% Triton X100, preabsorbed with 10% fetal calf serum, and incubated overnight at 4 °C with a combination of primary antibodies at 0.5 μg ml^−1^ each: mouse anti-ankyrin G (Catalogue sc-12719, Santa Cruz Biotech) and chicken anti-neurofilament-A (Catalogue AB5539, EMD Millipore). The membrane was subsequently incubated with 1:200 dilutions of anti-mouse IgG ALEXA488 (Catalogue A-11001, ThermoFisher), anti-chicken Ig ALEXA594 (Catalogue A-11042, ThermoFisher) and 1:1,000 TOPRO iodide solution (Catalogue T3605, ThermoFisher). Images of upper and lower membrane surfaces were captured with a Leica TCS2 confocal microscope.

### Brain slice immunofluorescence

Sagittal cryosections of paraformaldehyde submersion-fixed brain from adult WT mice were probed by immunofluorescence, as above, with different combinations of primary antibodies at 0.5 μg ml^−1^: mouse anti-FHF4 (N56/21), mouse anti-A-type FHF (N235/22), custom rabbit anti-FHF2 (ref. 31), pan-sodium channel (K58/35), and rabbit anti-CASPR (Catalogue ab34151, Abcam) followed by incubations with secondary antibodies conjugated to fluorophores ALEXA488, ALEXA594, or ALEXA643 (1:200 dilution) (ThermoFisher). When employing two primary antibodies from a common host species, each primary antibody and corresponding secondary antibody were incubated successively to ensure against primary antibody recognition by off-target secondary reagent. In some cases, nuclei were stained with TOPRO iodide. Confocal images were captured and images spanning 2–5 μm through the *z* axis were merged.

### Electrophysiology

Neurons on coverslips were visualized by video camera with infrared light and differential interference contrast optics from the recording chamber of a Nikon Eclipse FN1 microscope using a 63 × water-immersion lens and a 2 × optical magnifier in the light path. The cells were continuously perfused in carbogen-bubbled artificial cerebrospinal fluid (120 mM NaCl—26 mM NaHCO_3_—3 mM KCl—1.2 mM KH_2_PO_4_—3 mM D-glucose—2 mM Na pyruvate—3 mM myo-inositol—2 mM CaCl_2_—1.2 mM MgSO_4_). Cells were whole-cell patched with borosilicate pipettes pulled to resistance of 16–20 MΩ when filled with pipette solution (126 mM K gluconate—4 mM NaCl—5 mM HEPES—15 mM D-glucose—1 mM MgSO_4_—50 μM CaCl_2_—150 μM BAPTA—3 mM Mg ATP—100 μM GTP adjusted to pH 7.2 with KOH). Voltage clamp and current-clamp commands and recordings were made using a MultiClamp700 amplifier, Digidata1440 analogue/digital interface, and pCLAMP10 software (Molecular Devices). All recordings were conducted at 25 °C.

### Dynamic fluorescence in dye-filled GrC processes

All fluorescence imaging was conducted at 25 °C. Individual granule cells were filled with the amphiphilic voltage-sensitive dye JPW3028 (ref. 14). Patch pipettes were first back-filled with dye-free pipette solution and then filled with pipette solution containing 0.8 mM JPW3028. A neuron's soma was whole-cell patched and voltage clamped to −70 mV for 15 min to allow for passive diffusion of dye in the cell soma. The dye-containing pipette was carefully pulled off the cell generating an outside-out patch on the pipette tip while sealing the cell, and the cell was maintained one hour to allow for dye permeation of the axon. The cell was then repatched in the soma with a pipette containing dye-free solution and placed in voltage-clamp or current-clamp mode. Alternatively, a theta glass pipette coated with rhodamine-conjugated serum albumin and attached to a bipolar stimulus electrode was positioned under fluorescence guidance to a point along the distal axon. Fluorescence imaging of the neuron was performed using a SOLA-SE LED Light Engine (Lumencor), a NeuroCCD-SM high-speed camera (Redshirt Imaging), and a filter cube consisting of a 520±45 nm excitation interference filter, a dichroic mirror with 570 nm central wave length, and 610 nm barrier emission filter. The CCD camera was attached to a separate port on the microscope that contained a 0.1 × demagnifier to allow low-magnification imaging of a 280 μm diameter area with pixel dimension of 3.54 μm × 3.54 μm. Neuroplex software (Redshirt Imaging) controlled timing of the light source, triggered initiation of a voltage-clamp or current-clamp protocol, and image capture at either 2 or 5 kHz. Direct axonal stimulation at 60 Hz with 0.2 ms current pulses from the theta pipette were delivered using a Grass Stimulator and stimulus isolator unit also under the control of the Neuroplex software.

Voltage clamp step protocols were repeated for 30–50 trials, fluorescence data were captured at 2 kHz, and data from trials were averaged. In some experiments, voltage-gated currents were block by the adding 0.2 mM CdCl_2_, 5 mM 4-amino purine, 30 mM tetraethylamine and 1 μM tetrodotoxin (TTX) (Catalogue 1069, Tocris) to the extracellular solution and reducing the concentration of NaCl to maintain iso-osmolarity. Current-clamp protocols were used to induce action potentials. Single action potentials were generated with a 0.2 msec positive current pulse, which was repeated every 3 s for 30–50 trials while capturing images at 5 kHz. To achieve more accurate measurement of the conducting spike waveform, fluorescence data from all trials were averaged using the somatically recorded spike for alignment of data from each trial. To improve the signal-to-noise ratio of fluorescence signals, data from 3–5 neighbouring pixels were averaged. For 60 Hz current stimulations from either soma or distal axon, 10–15 trials were conducted with 2 kHz image capture rate. Somatic current injections were biphasic (700 pA 1 ms, −700 pA 1 ms). Biphasic pulses facilitated recovery from inactivation of soma-proximal sodium channels, allowing for repetitive firing even in *Fhf1*^−/−^ *Fhf4*^−/−^ neurons. Control experiments in the presence of voltage-gated channel inhibitors demonstrated that biphasic stimulation currents do not influence axon membrane voltage beyond 100 μm from the soma (Supplementary Fig. 6), showing that this stimulation protocol does not artificially bias measurements of repetitive spike conduction.

After completing a dynamic fluorescence experiment, cell fluorescence was photographed in a series of high magnification and resolution images (9.5 pixels per μm). The axon was then traced in graphics software as a 1-pixel-wide line, and axonal distances from soma were calculated from the pixels in the trace.

### Computational modelling of GrC excitation and conduction

The cell model bears the same number of compartments as described previously^10^, including four dendrites, soma, hillock, 56 axonal segments (2.3 μm length each), and 500 parallel fibre (PF) segments (10 μm length each) (Fig. 5a). The proximal three axonal segments (=6.9 μm length) are designated as the AIS. The axon and PFs are 150 nm diameter. The conductances in the model are described in Supplementary Table 2, with the exception of dendritic conductances, which are unchanged from previous description^10^.

We employed a 12-state Markov model for voltage-gated sodium channels, as diagrammed in the schematic below.


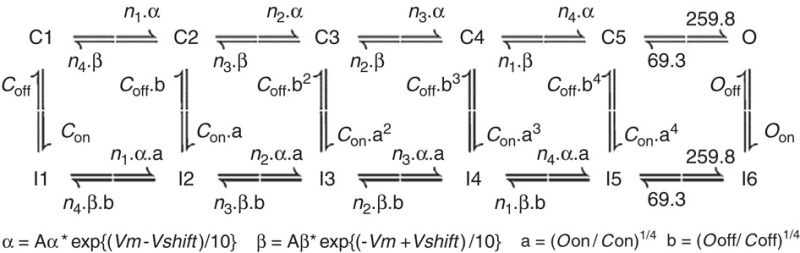


This model is an adaptation of the 13-state model of Khaliq *et al.*^23^, with the blocked open state and consequent resurgent current omitted. While resurgent current has a small effect on high-frequency firing in granule cells^36^, our omission was due to the incompatibility of even small resurgent current in the distal axon and parallel fibre on post-spike repolarization, due to the very low-distal leak conductance in our model. *n*_1_=5.422, *n*_2_=3.279, *n*_3_=1.83, *n*_4_=0.738, as in the previous version of the channel model^4^. Rate parameters *A*_α_, A_β_, *C*_on_, *C*_off_, *O*_on_, *O*_off_, and *V*_shift_ are listed in Supplementary Table 2 and were differentially set to values that simulated the voltage-dependent and kinetic properties of channels either in the absence or presence of associated FHF proteins (Supplementary Fig. 5) in approximate agreement with somatic recordings of sodium currents in WT and *Fhf1*^−/−^*Fhf4*^−/−^ granule cells^4^ (see Supplementary Table 1 for comparisons). The maximal sodium conductance in the parallel fibres (110 mS cm^−2^) corresponds to an estimated 250–300 channels per 10 μm segment, assuming a single-channel conductance of 14–20 pS^37^,^38^.

We note that in comparison to the original Na_v_ model of Khaliq *et al.*^23^, the Na_v_ model here has comparable voltage dependence of activation but displays slower activation at negative potentials near or below *V*_1/2_ activation (Supplementary Fig. 5G). These kinetics are a better fit to recorded sodium currents in granules cells^4^ (Supplementary Fig. 5G) or in transfected cells expressing Na_v_1.6 (ref. 9). While slowing Na_v_ activation/deactivation kinetics impacts excitability, it does not significantly affect Na_v_ currents associated with spike conduction, which is primarily affected by Na_v_ open-state inactivation dictated by the rate constant *O*_on_.

In comparison to the previously used model for delayed rectifier potassium channel (K_v_)^4^, the revised K_v_ model has 4.5-fold slower activation and deactivation rate constants (Aalpha_n=−0.00222, Abeta_n=0.0285), while preserving the same voltage dependence of activation. This modification to K_v_ kinetics was crucial for achieving realistic simulations of conducting action potentials by delaying the spike K_v_ current onset relative to the depolarizing Na_v_ current and by delaying K_v_ deactivation, facilitating the post-spike return of axonal and parallel fibre membrane to resting potential. Even a twofold faster K_v_ kinetics than employed here increases the temporal overlap between spike Na_v_ and K_v_ currents, resulting in substantially narrower spikes inconsistent with voltage-sensitive dye recordings and increasing current flux per spike several-fold.

In order to test the importance of FHF exclusion from sodium channels beyond the AIS, the parallel fibres were remodelled to bear FHFs while maintaining the waveform of the conducting action potential. Parameters for the PFs were changed as follows: *C*_on_=0.003464, ms^−1^, *C*_off_=0.4157, ms^−1^, *O*_on_=1.8186, ms^−1^, *O*_off_=0.006928, ms^−1^, *V*_shift_=−22 mV, 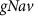
=13.5 mS cm^−2^, 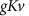
=18.5 mS cm^−2^.

Current-clamp simulations of spike initiation and conduction were performed by injecting a continuous current into the soma. During simulations, membrane voltage and ionic currents in axonal compartments were monitored. Spike-associated currents were used to calculate ion fluxes per spike, which together with dimensions of the axon and parallel fibre and the relationship of charge to monovalent ion number (1 C=10 μmol ions) enabled calculation of changes in ion concentration within the cell process. To determine the theoretical minimum charge transfer needed to raise the voltage from −70 to +60 mV, we assume that all inward sodium current serves to charge the membrane with no inward current wasted to countervailing outward ionic current or axial current. A parallel fibre 10 μm segment of 150 nm diameter has volume of 1.77 fl and capacitance of 47 fF. To achieve a Δ*V* of 130 mV, Δ*Q*=*C* × Δ*V*=6.12 fC, which is an intracellular ion concentration of 0.35 mM.

The passive charging along an axon in response to a somatic voltage step is influenced by the axon's axial resistance, leak conductance, diameter, and length. In order to compare modelling simulations to passive charging measurements obtained using voltage-sensitive dye, the cell model's axonal length required adjustment to those of cultured neurons. Based on the measured length of several cultured granule cell axons filled with cadaverine-ALEXA488 conjugate (1.31±0.17 mm, *n=4*), the cell model was adjusted to bear an unbranched axon of 1.2 mm length. Simulations were conducted by application of a 10 mV hyperpolarizing voltage step to the soma, and membrane voltages at different points along the axon were monitored. The model axon's leak, diameter and axial resistance were manipulated to give passive charging simulation in agreement with fluorescence empirical measurements.

All simulations were performed with the NEURON simulation software^39^.

### Data availability

GrC model files will be made available for download at https://senselab.med.yale.edu/modeldb/.

## Additional information

**How to cite this article:** Dover, K. *et al.* FHF-independent conduction of action potentials along the leak-resistant cerebellar granule cell axon. *Nat. Commun.* 7:12895 doi: 10.1038/ncomms12895 (2016).

## Supplementary Material

Supplementary Information Supplementary Figures 1-6, Supplementary Tables 1-2 and Supplementary References.

Supplementary Movie 1 **Initiation and conduction of an action potential in a cultured wild-type granule cell.** A JPW3028-filled granule cell was patched and stimulated by injection with a 2 nA 0.2 msec current pulse. Fluorescence was imaged at 5 KHz, and data from 40 stimulation cycles at 0.2 Hz were averaged. The color scale represents normalized light intensity (I/RLI) at each pixel ranging from 1.00 (dark violet) to 1.07 (white). The spike initiates at the AIS prior to the electrically recorded somatic action potential peak (inset) and conducts down axon, bifurcating at axonal branch points. Fluorescence change in the two dendrites is much less than detected in the axon owing to greater association of dye with internal membranes in the wider dendritic processes. The vast excess of internal dye in the soma precludes fluorescence dynamics in this compartment. Scale: 1 pixel = 3.5 μm.
