# Supplementary material for: FHF-independent conduction of action potentials along the leak-resistant cerebellar granule cell axon
Source: Nat Commun. 2016 Sep 26;7:12895. doi: 10.1038/ncomms12895 (PMC5052690; doi:10.1038/ncomms12895)
Supplement: Supplementary Information — Supplementary Figures 1-6, Supplementary Tables 1-2 and Supplementary References. [file ncomms12895-s1.pdf]

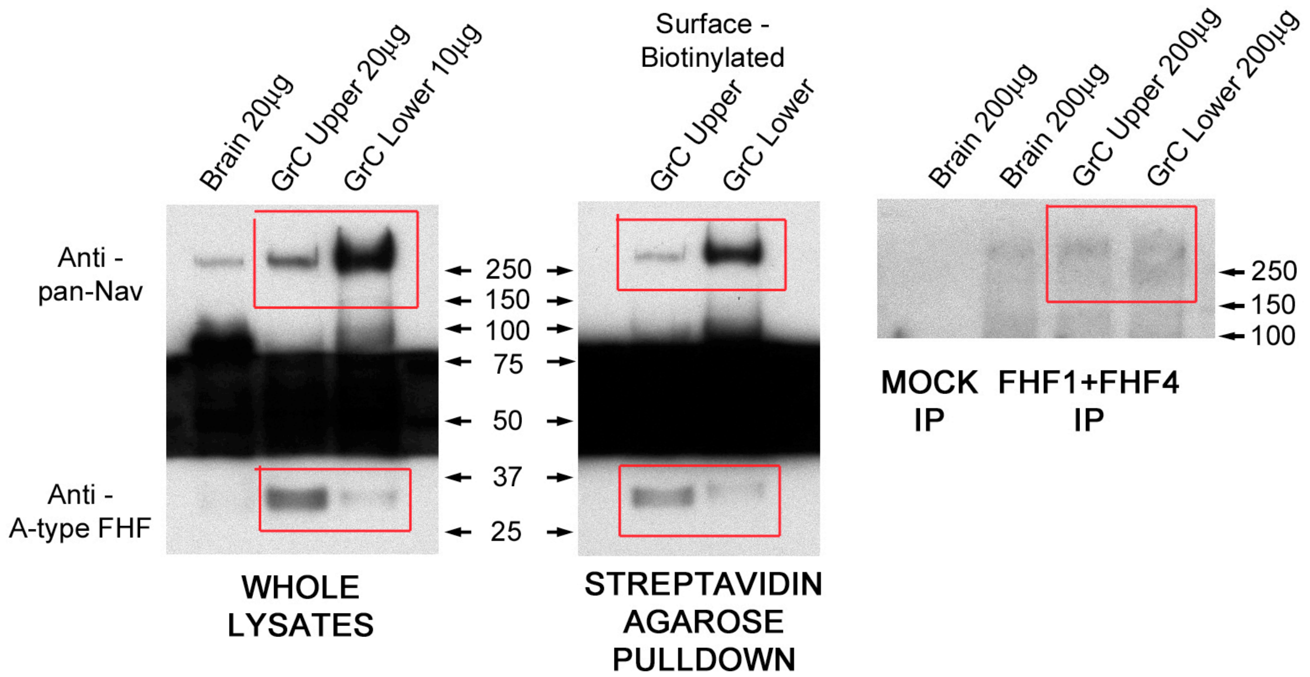

Supplementary Figure 1. Immunoblot analysis of FHF, Na_V, and FHF/Na_V complexes in lysates of total cells and distal axons. The left, center, and right images represent the uncropped ECL film exposures from which cropped sections (framed in red) are present in Figure 1D. *Left,* protein extracted from whole brain (20 µg), upper surface (whole cell) of GrC hanging culture (20 µg), and lower surface (distal axons) of the same hanging culture (10 µg) were directly electrophoresed and immunoblots cut into horizontal strips for probing with monoclonal anti-pan-Na_V (high molecular weight strip), an irrelevant antibody (middle strip), and monoclonal anti-A-type FHF (low molecular weight strip). Following application of peroxidase-conjugated secondary antibody and enhanced chemiluminescence (ECL) reagent, the filter strips were reassembled before film exposure. The middle strip yielded high background. Data show that the A-type FHF: Na_V ratio is at least 5-fold lower in the GrC distal axons compared to GrC whole cells. *Center,* live GrC hanging cultures were surface-biotinylated before extraction and streptavidin-agarose pull-down of labeled proteins, which were electrophoresed and immunoblotted in strips as described above. FHF/Na_V ratios demonstrate that only a small fraction, at most, of plasma membrane Na_V is associated with cytoplasmic A-type FHF on GrC distal axons. *Right,* equal amounts of whole cell (upper surface) and distal axon (lower surface) GrC lysates were immunoprecipitated with a combination of antibodies specific for all isoforms of FHF1 and FHF4 followed by immunoblot detection of sodium channels. A much smaller fraction of sodium channels in distal axons show association with FHF1 and FHF4 isoforms (compare band intensities to those in left panel). Marker sizes are indicated in kilodaltons.

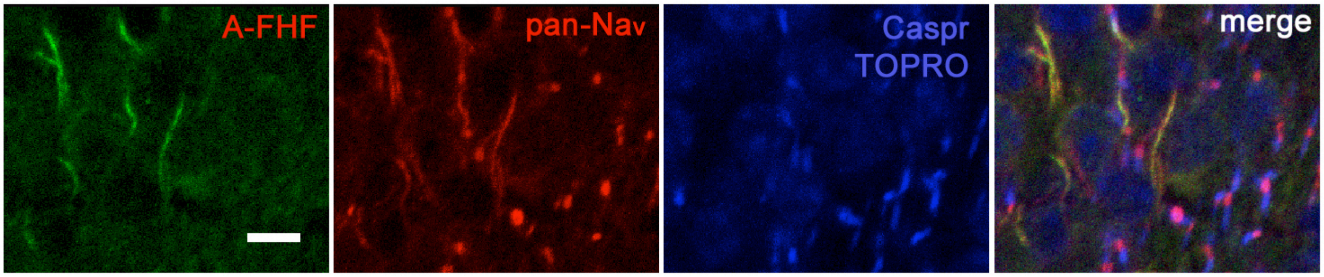

Supplementary Figure 2. A-type FHF localization in cerebellum. Immunofluorescence detection of A-type FHFs (green), sodium channels (red), and Caspr (blue) are shown in a high-magnification section of cerebellar cortex that includes granule layer (left side of image) and adjacent white matter (right side). Axon initial segments in the granule layer are positive for A-type FHF, while white matter nodes of Ranvier bear Na_V and flanking Caspr in the absence of A-type FHF. Nuclei were visualized with TOPRO iodide (blued). Scale bar: 4 μm.

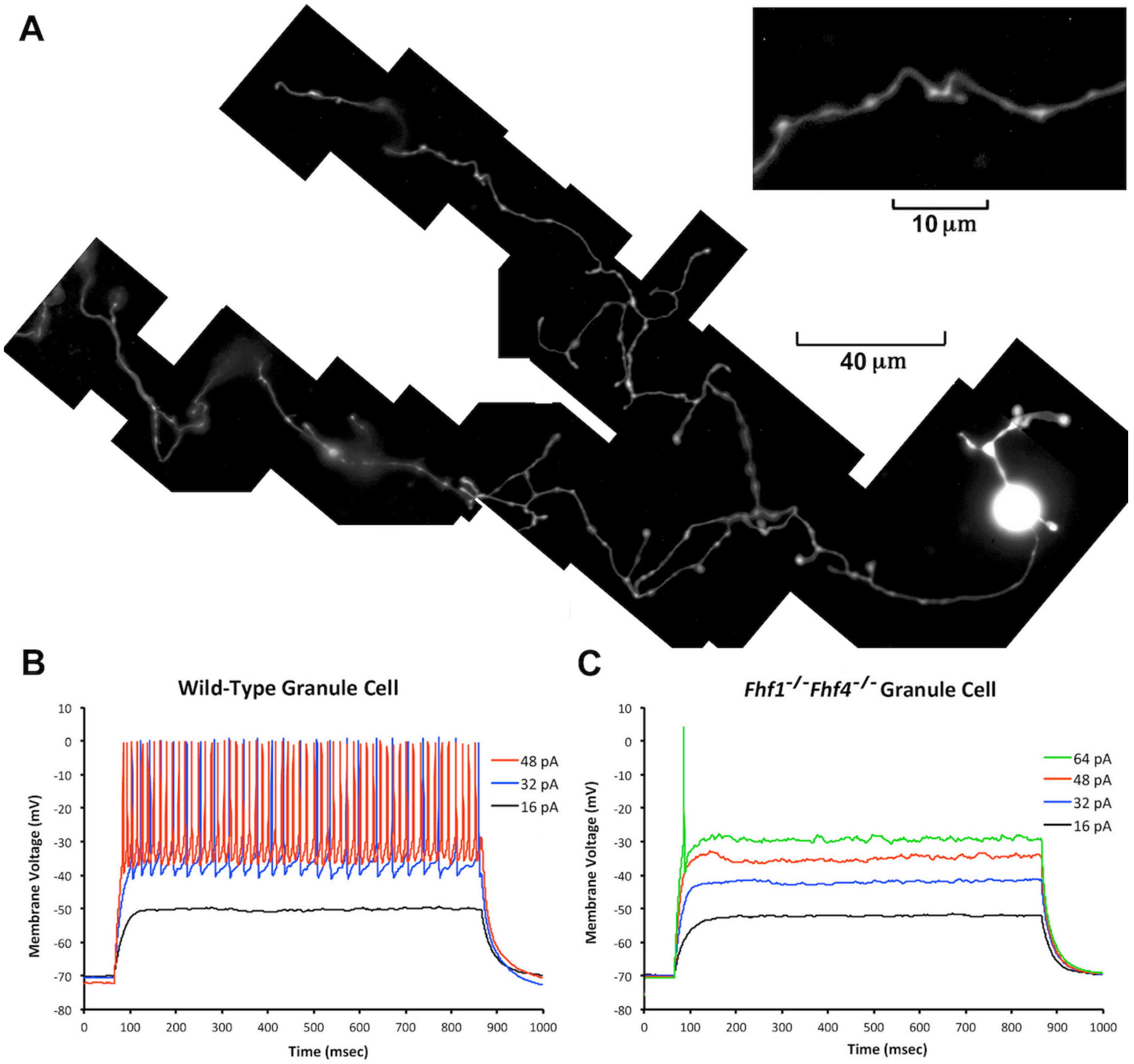

Supplementary Figure 3. Morphology and excitability of cultured GrCs. A) Morphology of a cultured granule neuron. A neuron was whole-cell patched with a pipette containing cadaverine-ALEXA488 conjugate and live images captured after 15 minutes. The neuron contains two short brightly fluorescing dendrites and a long axon of 1 mm aggregate length with a major bifurcation, several smaller branches, and varicosities spaced on average ~5 μm apart. Some varicosities likely represent mitochondria bulges [1]. B) Excitability of a wild-type cultured granule cell. A patched neuron was injected for 800 msec with different amplitudes of depolarizing current. The cell responds with action potential trains that increase in frequency as current is increased, comparable to the behavior of granule neurons recorded from cerebellar slices [2]. C) Excitability of a *Fhf1*$^{-/-}$*Fhf4*$^{-/-}$ cultured granule cell. Current injection only triggers an initial spike without continued firing, comparable to behavior of mutant granule neurons recorded from cerebellar slices [3].

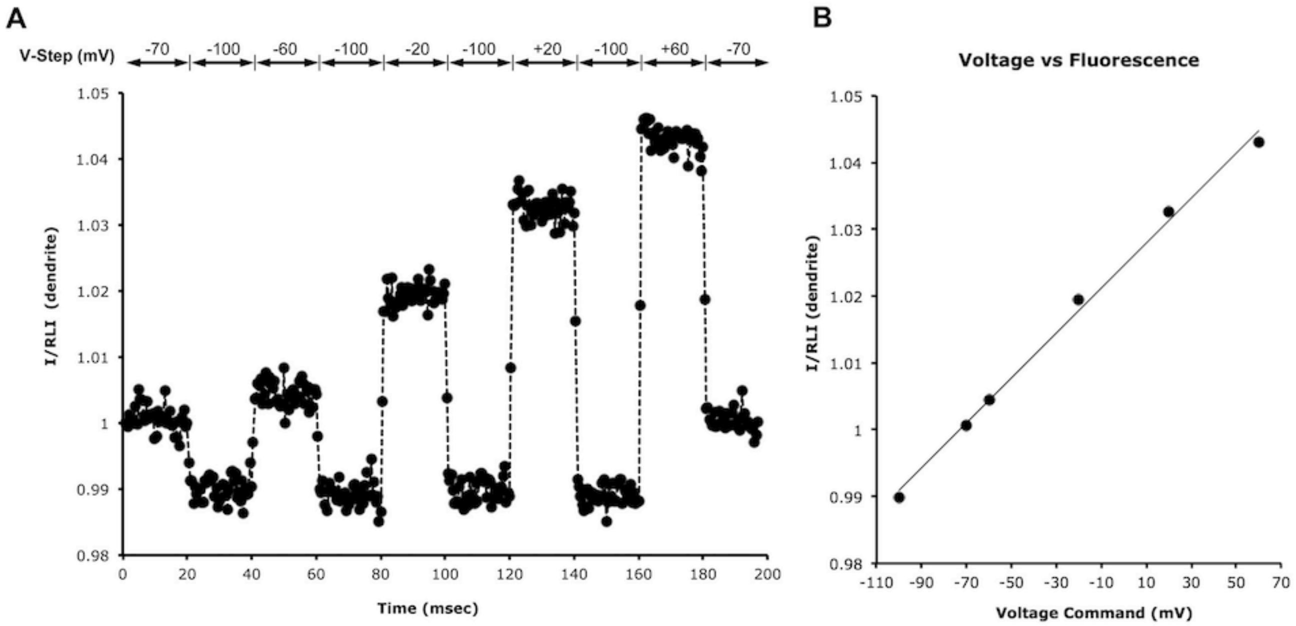

Supplementary Figure 4. Linear relationship of sensor fluorescence and membrane voltage in a JPW3028-filled GrC. A cultured neuron previously filled with fluorescent voltage sensor JPW3028 was patched and subjected to voltage-clamped voltage steps in the presence of voltage-gated channel inhibitors, and fluorescence in the proximal dendrite (<30 μm from soma) was sampled at 0.5 msec intervals. A) Plot of the ratio of fluorescence intensity (I) to resting light intensity (RLI) over time. Voltage step commands are indicated above graph. B) Plot of I/RLI versus membrane voltage command shows a linear relationship from -100 mV to +60 mV.

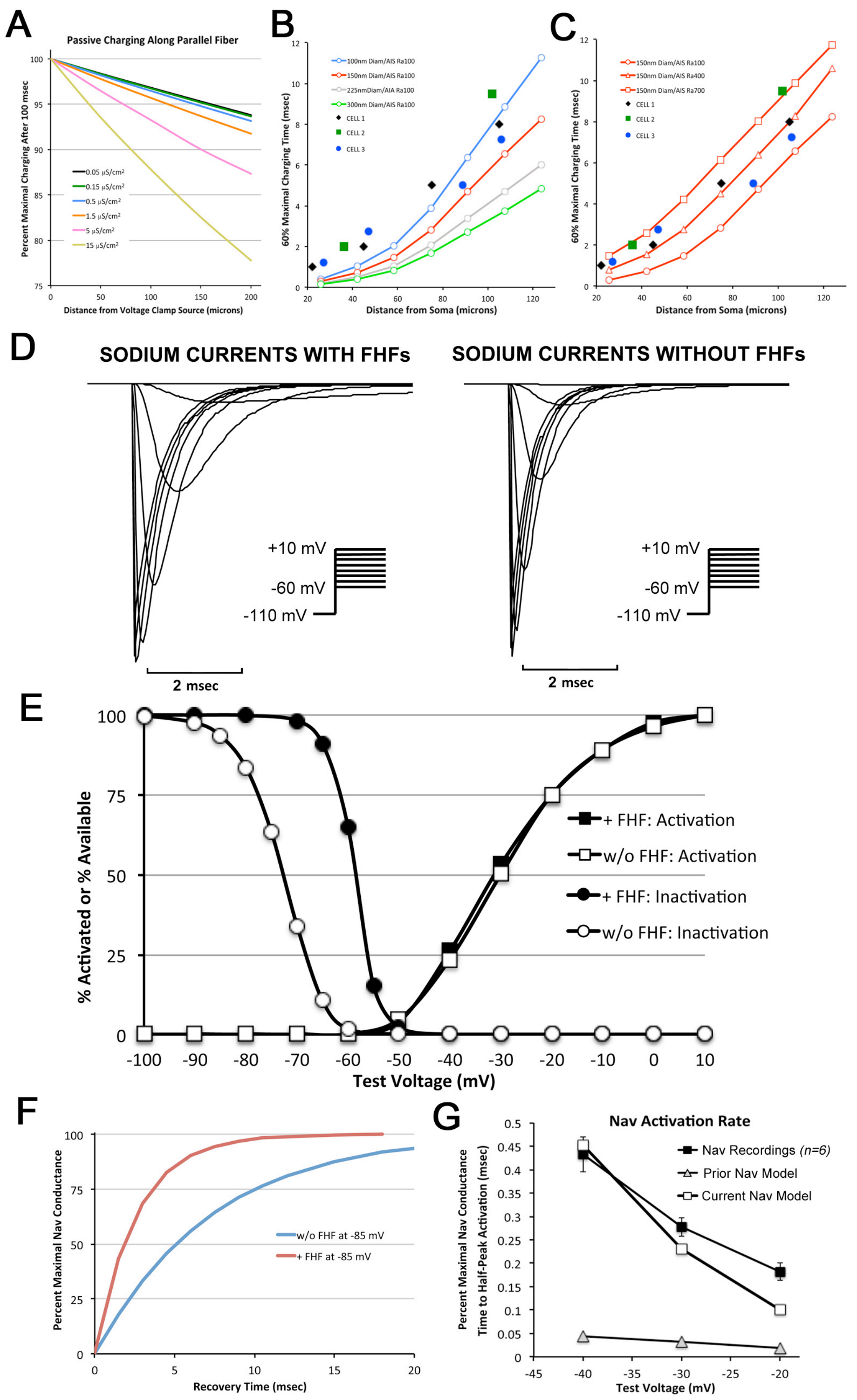

Supplementary Figure 5. Simulations of axonal passive properties and sodium channel gating. A) Passive charging along the axon and parallel fiber. Leak conductance density along the 150 nm axon and parallel fibers were varied from 0.05 μS/cm² to 15 μS/cm² and, for each leak value, a somatic voltage-clamped step from-70 mV to -80 mV was simulated and voltages at different distances from soma

monitored after 100 msec. Data are expressed as the percentage of maximal (-10 mV) voltage change versus distance from soma. Charging is affected by less than 10% at fiber conductance densities up to 5 $\mu S/cm^2$, but falls off at higher leak conductance values. As voltage-sensitive fluorescence changes induced by somatic voltage steps applied to cultured neurons does not fall off more than 10% over distances up to 200 μm, axon and parallel fiber leak conductance in the model was set at 5 $\mu S/cm^2$ for further simulations. B) Passive charging rate along the axon and parallel fibers. The axoplasmic resistance (R_a) was set at standard value of 100 Ω*cm, and the diameter of the axon and parallel fibers was set to either 150, 300, or 400 nm. In response to a -10 mV somatic voltage step, the time until 60% maximal charging was monitored at different distances from soma. None of these simulations gave acceptable values for charging times in the proximal region of axon when compared to fluorescence measurements for three cultured neurons (circles). C) Elevated axial resistance in proximal axon improves simulation of passive charging kinetics. Axial resistance (R_a) in the axon initial segment was set to either 100, 400, or 700 Ω*cm, and charging kinetics along the 150 nm axon and parallel fibers was monitored during simulated somatic voltage step. Elevated proximal axial resistance in the granule cell model yielded simulations with better fit to fluorescence data (circles). D – F) Voltage clamp simulations of sodium currents modeled with or without associated FHFs. The parameters for the sodium channel model with or without FHFs are described in Materials and Methods and in Supplementary Table 2. Voltage dependence of transient sodium current (D), voltage dependence of sodium conductance activation and steady state inactivation (E), and repolarization-induced recovery rate from inactivation at -85 mV (F) all give values in approximate agreement with somatically recorded sodium currents from wild-type (with FHF) and *Fhf1*$^{-/-}$*Fhf4*$^{-/-}$ (without FHF) cultured cerebellar granule cells [3] (see Supplementary Table 1). G) Na_V activation rate. The time for sodium current rise to half-maximal activation is plotted vs. test voltages of -40, -30, and -20 mV. The current version of the Na_V model (with or without FHF) gives activation times similar to those recorded in cultured cerebellar granule cells [3], while the earlier described Na_V model [4] activated up to 10-fold faster.

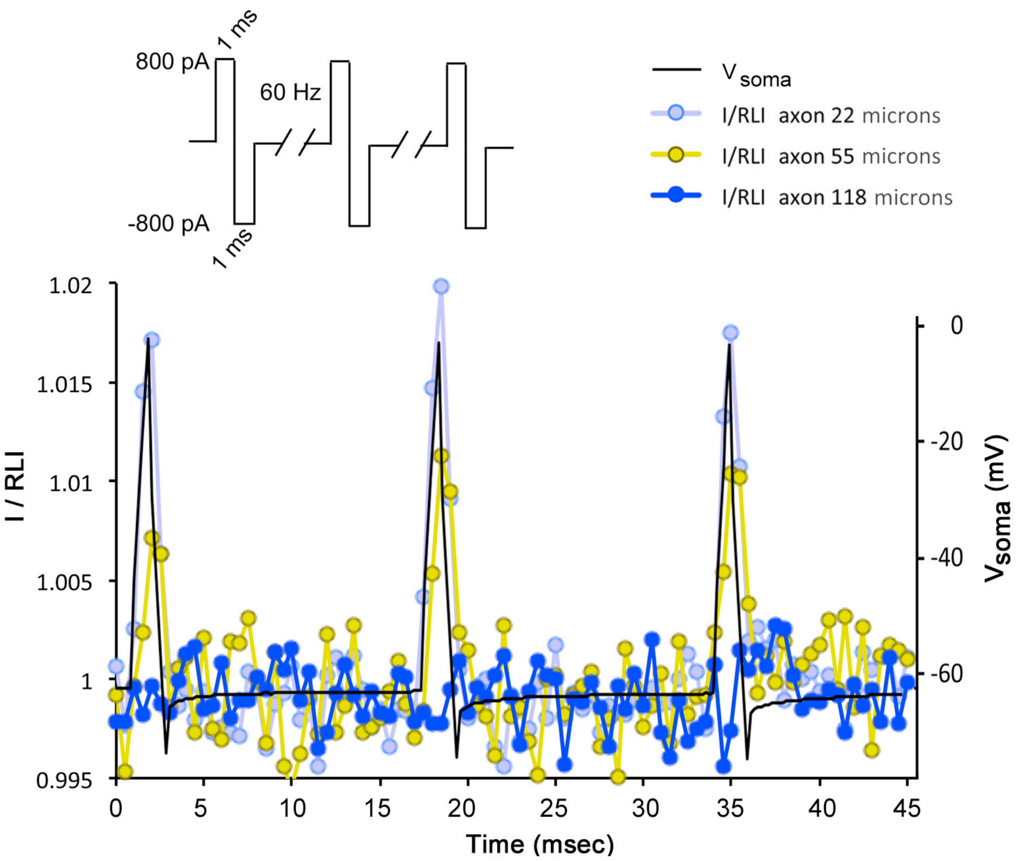

Supplementary Figure 6. Passive response of the GrC axon to somatically injected currents. A cultured neuron previously filled with fluorescent voltage sensor JPW3028 was patched and subjected to 60 Hz biphasic somatic current pulses as indicated atop figure in the presence of voltage-gated channel inhibitors while recording somatic membrane voltage and fluorescence intensity along the axon. While current pulses induced fluorescence transients in the proximal axon, no fluorescence change was detectable on the axon beyond 120 μm. This finding shows that biphasic pulses can be used to artificially enable repetitive spike initiation in the proximal axon, with spike conduction down the distal axon (Figure 3G,H) driven solely by intrinsic ionic mechanisms.

Parameter	FHF WT Na_V Model	FHF KO Na_V Model	FHF WT Na_V Recording	FHF KO Na_V Recording
$V_{1/2}$ Inactivation (mV)	-58.7	-72.8	-59.1	-72.8
Tau Inactivation at -5 mV (msec)	0.60	0.40	0.64	0.33
Tau Recovery -85 mV (msec)	2.6	7.3	3.8	5.9
$V_{1/2}$ Activation (mV)	-30.8	-30.1	-33.3	-36.5
Time to 50% Activation at -40 mV (msec)	0.45	N.T.	0.43	N.T.

Supplementary Table 1. Comparison of parameters for Na_V model and GrC Na_V recordings. The table lists values for several Na_V parameters derived from model simulations and previously reported recordings from cultured cerebellar granule neurons [3]. N.T., not tested.

Conductance	Soma	Hillock (with FHF)	AIS (with FHF)	Distal Axon (w/o FHF)	PFs (w/o FHF)
$Na^+(Na_V)$					
$\overline{g_{Nav}}$ (mS/cm^2)	0	130	750	330	110
A_α (ms^{-1})		8.85	8.85	8.85	8.85
A_β (ms^{-1})		0.0318	0.0318	0.0318	0.0318
V_{shift} (mV)		-22	-22	-25	-25
C_{on} (ms^{-1})		0.003464	0.003464	0.09353	0.09353
C_{off} (ms^{-1})		0.4157	0.4157	0.04676	0.04676
O_{on} (ms^{-1})		1.8186	1.8186	2.5981	2.5981
O_{off} (ms^{-1})		0.006928	0.006928	0.000173	0.000173
K^+ (K_V)					
$\overline{g_{Kv}}$ (mS/cm^2)	0	3,000	10,000	44	13
K^+ (K_{IR})					
$\overline{g_{KIR}}$ (mS/cm^2)	2	0	0	0	0
K^+ (K_M)					
$\overline{g_{KM}}$ (mS/cm^2)	6	0	0	0	0
K^+ (K_A)					
$\overline{g_{KA}}$ (mS/cm^2)	40	0	0	0	0
Leak $gLKG1$ (mS/cm^2) (e_{LKG1} = -16.5mV)	0.0916	0	0	0	0
Leak $gLKG3$ (mS/cm^2) (e_{LKG3} = -74.5 mV)	0	0	2	0.005	0.005

Supplementary Table 2. Conductances for the cerebellar GrC model at 25°C. Conductances in somatic, hillock, AIS, distal axon, and parallel fibers (PFs) are shown, along with gating parameters for the sodium conductance. Sodium conductance in the hillock and AIS are modeled as influenced by FHF/Na_V association, while sodium conductance in distal axon and PFs are modeled in absence of FHF influence (see simulations in Supplementary Fig. 5). Dendritic conductances are not indicated, and are unchanged from the granule cell model described previously [4].

Supplementary References

1. Xu-Friedman, M. A., Harris, K. M., Regehr, W. G. Three-dimensional comparison of ultrastructural characteristics at depressing and facilitating synapses onto cerebellar Purkinje cells. *J. Neurosci.* **21,** 6666-6672 (2001).

2. D'Angelo, E., De Filippi, G., Rossi, P., Taglietti, V. Synaptic excitation of individual cerebellar granule cells in situ: evidence for the role of NMDA receptors. *J. Physiol.* **484,** 397-413 (1995).

3. Goldfarb, M. *et al*. Fibroblast growth factor homologous factors control neuronal excitability through modulation of voltage-gated sodium channels. *Neuron* **55,** 449-463 (2007).

4. Diwakar, S., Magistretti, J., Goldfarb, M., Naldi, G., D'Angelo, E. Axonal Na^+ channels ensure fast spike activation and back-propagation in cerebellar granule neurons. *J. Neurophysiol.* **101,** 519-532 (2009).
